# Early predictors of poor treatment response in patients with schizophrenia treated with atypical antipsychotics

**DOI:** 10.1186/s12888-018-1950-1

**Published:** 2018-12-04

**Authors:** Yi-Lung Chen, Kun-Po Chen, Chih-Chiang Chiu, Ming-Hong Tai, For-Wey Lung

**Affiliations:** 1 Department of Biological Sciences, National Sun Yat-sen University, Kaohsiung, Taiwan; 2 Department of Psychiatry, Taipei City Psychiatric Center, Taipei City Hospital, Taipei, Taiwan; 3 Department of Psychiatry, School of Medicine, College of Medicine, Taipei Medical University, Taipei, Taiwan; 4 Instituteof Biomedical Sciences, National Sun Yat-Sen University, 70 Lienhai Rd, Kaohsiung, 80424 Taiwan, Republic of China; 5 Calo Psychiatric Center, No.12-200, Jinhua Rd., Xinpi Township, Pingtung County 925 Taiwan; 6 Graduate Institute of Medical Sciences, National Defense Medical University, Taipei, Taiwan

**Keywords:** Schizophrenia, PANSS, Positive predictive value, Negative predictive value

## Abstract

**Background:**

The aims of this study were to explore the relationship between early reduction in psychotic symptoms and the ultimate response in patients with schizophrenia treated by atypical antipsychotics, and to determine the best time to switch or maitain the regimen. We also explore the possible predictors for the clinical response.

**Methods:**

One hundred eleven inpatients with acutely exacerbated schizophrenia were randomized to give optimal therapy of olanzapine, risperidone, and paliperidone in one-week run-in period and 12 weeks’ intervention. All participants were assessed using Positive and Negative Syndrome Scale (PANSS). Early Response, defined as reduction of 25% in PANSS score, was examined at weeks 1, 2, 3, 4 and 8, and these ratings were used to predict ultimate response (25% PANSS reduction) at week 12. We hypothesized that early treatment response at Week 1 or 2 could predict Week 12’s treatment outcome.

**Results:**

The early treatment response at Week 2 had a greater negative prediction value (NPV, 93.6%) than did the response at Week 1 (NPV, 69.7%), Week 3 (NPV, 91.5%), Week 4 (NPV, 90.7%) and Week 8 (NPV, 87.2%). The positive predictive value became more acceptable (65%) until Week 4. There was no any other potential predictors, including types of antipsychotics medication and treatment dosage, were associated with ultimate response in this study.

**Conclusion:**

The treatment non-response at Week 2 optimally predicted the ultimate (Week 12) non-response, in terms of negative predictive value (NPV). These finding suggests that the revision of treatment strategy should be considered t if patients with schizophrenia was not responsive to them after 2 weeks’ treatment, and for those who are responders at Week 2, another two weeks are needed to further evaluate whether they will be continuously responsive.

**Trial registration:**

NCT03730857 at ClinicalTrial.gov. Date of registration: 30/Oct/2018.

## Background

Schizophrenia is considered to be the most complex and diverse form of mental disorder. Although the response of treatment in schizophrenia varied, early identification of treatment response in these patients is considered vital in prediction of the efficacy of antipsychotic treatment [1]. Experiments have tried to discriminate the responders and poor responders as early as possible and to assess prognostic factors before and/or during treatment [1, 2]. These results provide insight into methods of individualized prediction.

However, the pretreatment discrimination of responders from poor responders remains unsatisfactory to a large extent. In this context, the predictor variables may potentially affect the treatment outcome of schizophrenia. There have been several controversies regarding the role of predictors in relation to the final response to therapy in patients with schizophrenia [2]. Agidet al. [3] have conducted meta-analyses in patients with schizophrenia or schizoaffective disorder and proposed that the response to antipsychotic therapy started from the first week of treatment and increased over time. There is a need to identify a lack of response to antipsychotics early in the course of treatment in order to prevent unnecessary persistence with unreliable agents, lessen the risk of adverse events, minimize the duration of hospitalization, and reduce the cost and burden of illness [4, 5]. Previous study has shown that the possibility of predicting antipsychotic response early in the course of treatment in hospitalized schizophrenic patients and provided the best combination of sensitivity, specificity, and positive and negative predictive values on predicting treatment outcome [6]. A recent diagnostic test meta-analysis with 34 studies and 9460 participants conducted by Samara et al. examined whether non-improvement at week 2 could later predict non-response to antipsychotics in patients with schizophrenia. Some evidence indicated that the lack of treatment improvement at week 2 predicted non-response at endpoint with a specificity of 86% and a positive predictive value (PPV) of 90% (nonresponse at endpoint) [7].

Recently, there has been increased research interest in the association between the reduction in psychotic symptoms during the early stages of treatment and the prediction of later treatment response [2, 5, 8–11]. According to the treatment guidelines of the American Psychiatric Association for the better management of patients with schizophrenia, health care professionals should monitor the response to antipsychotic therapy for 2–4 weeks prior to increasing the dose or changing the medication [12]. However, the precise time course of the clinical improvement with antipsychotic treatment needs to be established.

There is limited information on the response rates of patients with schizophrenia to antipsychotic drugs and unsatisfactory availability of clinical predictors of outcome status in these patients. Here, we present a prediction algorithm that uses the early response to antipsychotics, coupled with relevant patients’ characteristics, to forecast the subsequent response in the treatment of patients with schizophrenia. The aim of this 12-week open-labeled study was to investigate whether early phase treatment response improvement in PANSS score reduction on weeks 1, 2, 3, 4 and 8 after atypical antipsychotics treatment in schizophrenic patients could predict their therapeutic outcome at Week 12. We would like to establish the best time to switch or maintain the regimen. In addition, we also explored the possible clinical predictors for the later clinical response.

## Methods

### Participants

This study recruited hospitalized adult patients who had a relapse of schizophrenia. All participants had received antipsychotic treatment for a period of time previously. They have to meet the diagnostic criteria for schizophrenia according to the Diagnostic and Statistical Manual of Mental Disorders, Fourth Edition (DSM-IV) [13]. One hundred and twenty adult inpatients were recruited and were randomly assigned to receive olanzapine (*n* = 60), risperidone (*n* = 30) and paliperidone (n = 30) in an allocation ratio of 2:1:1. The participants were allowed to change the dosage of antipsychotics and their hospitalization status according to the judgement of in-charged physicians during the study period.

The study was approved by the Institutional Review Board and written informed consents were obtained either directly from the patients or from their legal guardians after the study had been explained. The inclusion criteria for this study were: (1) age 18 to 65 years, (2) no major systemic illnesses based on physical examinations and laboratory test results, and (3) baseline PANSS total score≧60 [1]. The exclusion criteria were as follows: (1) participants not taking any antipsychotics in the previous one month, (2) participants were pregnant and lactating women, and (3) history of clozapine treatment in the previous 3 months, and (4) patients receiving long-acting antipsychotic injections in the preceding 6 months of enrollment.

### Study design

This study was conducted by a 12-week, open-label and naturalistic randomized design. A tri-therapy (olanzapine, risperidone, and paliperidone) completely randomized design was adopted for this study, which involved three homogeneous groups of patients with a run-in period of 3 months. The patients were required to have discontinued all prior use of antipsychotics for a period of at least 7 days before their entry into the study. During wash-out period, administration of either oral benzodiazpines, hypnotics or injection of lorazepam to control anxiety, insomnia and aggression were allowed. After the wash-out period, the patients received treatment with an atypical antipsychotic drug, olanzapine, risperidone or paliperidone for 3 months.

The Positive and Negative Syndrome Scale (PANSS) [14] rating scale was used to evaluate the changes of psychiatric symptoms in each time point. The subject patients were interviewed for the PANSS by senior psychiatrists. The raters in the present study had worked mainly on inpatient treatment of severe schizophrenic patients, and were well acquainted with symptoms of schizophrenia. Prior to the present study, all participating psychiatrists had received adequate training through the manual and they had had clinical experience in the PANSS rating before the study. At each time point if the scores of PANSS showed the patient’s symptoms had worsened, the dosage would be adjusted based on the clinical judgment of in-charged senior psychiatrist. However, if the scores of PANSS were improved, the dosage was maintained. The recommended dose for the three groups were as follows: 10 to 20 mg daily for olanzapine, 4 to 6 mg daily for risperidone, and 6 to 12 mg daily for paliperidone. Throughout the study period, the paticipants were allowed to continuously use some concomitant medication, including lorazepam (up to 6 mg/day) for insomnia or agitation and biperiden (up to 6 mg/day) for treatment of extrapyramidal side effects. No other psychotropic agents were administered during the 12-week study.

The participants were assessed by a senior psychiatrist who was familiar with the PANSS ratings at the initial meeting (day 0; Time 0), and were followed up and assessed at Week1, 2, 3, 4, 8, and 12 (Times 1–6). An structured interview for demographic and clinical data were obtained from each patient at the screening visit and a physical examination was performed. The symptoms of psychopathology were rated using the PANSS. The PANSS includes a total of 30 items that measure positive symptoms, negative symptoms, and general psychopathology [15]. The PANSS scores ranged from 30 to 210; higher scores indicated more severe psychopathology.

For treatment response, a variety of cut-offs (e.g. at least 20, 30%,40% or 50%) with regard to percentage reduction of the baseline scores of PANSS have been applied. Leucht et al. have revealed that a 25% Brief Psychiatric Rating Scale (BPRS) /PANSS reduction approximately means minimal improvement in terms of the Global Impressions (CGI) scale, and 50% reduction corresponding to much improved [16]. The definition of treatment response at each time point of the study period was established as 25% improvement in the PANSS total score. We conducted more frequent assessment time points (Time 0–7), which allowed us to identify the minimal improvement and decide a more accurate treatment strategy. The percentage of total PANSS score reduction was calculated as (PANSS_baseline_-PANSS_endpoint_/PANSS_basline_-30)*100% [16].

### Statistical analysis

The SPSS 21.0 software package (SPSS Inc., Chicago, IL) was used for the demographic, descriptive, exploratory and chi-square analyses, an1d Student’s t-test was used to discriminate the significant inter-group differences. We employed binary logistic regression to determine whether the treatment response at Week 1, Week 2, Week 3, Week 4 and Week 8 could predict the treatment responses at Week 12. We also calculated the sensitivity, specificity, positive predict value (PPV), negative predict value (NPV) and accuracy for each treatment response at weeks 1, 2, 3, 4 and 8, in terms of predicting the treatment outcome at Week 12.

We further analyzed the relationship between the potential predicators, the baseline clinical or demographic variables, and the final treatment outcome. The generalized estimating equations (GEE) [17] methodology was used to analyze longitudinal and other correlated data. The variables for predicting the final treatment outcome may be correlated, and thus GEE method was used to analyze the repeated measurement data. In the final GEE model, types of antipsychotics, treatment dosage, gender, age, age at onset, duration of mental illness, duration of the prior treatment, educational level, history of alcohol use and smoking, antipsychotics used before screening and family history of schizophrenia were treated as independent variables, with the final treatment response at Week12 as the dependent variable. In all analyses, *p* < 0.05 was taken to indicate statistical significance.

## Results

All participants’ clinical characteristics at baseline were comparable between these treatment groups.The demographic distribution of the participants is shown in Table 1. One hundred twenty eligible patients (olanzapine group consisted of 60 patients, risperidone group 30 patients, and the paliperidonegroup 30 patients) agreed to participate at the beginning of this study. Nine participants presented severe psychotic symptoms and made the washout clinically unfeasible. At the end, 111 participants were enrolled in this study: 53 participants (33 male and 20 female) with a mean age of 35.60 (SD 11.26) years treated by olanzapine, 28 participants (18 male and 10 female) with a mean age of 35.04 (SD 9.85) years treated by risperidone, and another 30 participants (21 male and 19 female) with a mean age of 36.21 (SD 11.27) years treated by paliperidone. There were no statistically significant differences between groups in terms of the baseline demographic and clinical characteristics. Seventy-one of 111 (63.9%) participants completed the 3-month study. The dropout rate of olanzapine treatment group was 47.2%, the risperidone treatment group 10.7%, and the paliperidone treatment group was 40.0%, respectively. All those who dropped out of the study had received treatment at least more than one month.

**Table 1:** Demographic and clinical characteristics of patients with schizophrenia treated with olanzapine, risperidone or paliperidone

	Olanzapine (*n* = 53)	Risperidone (*n* = 28)	Paliperidone (*n* = 30)	*p*
Male, N((%)	33 (62.3)	18 (64.3)	20 (66.7)	0.92
Age, mean ± SD (years)	35.60 ± 11.26	35.04 ± 9.85	36.21 ± 11.27	0.78
Age of onset, mean ± SD(years)	26.70 ± 8.46	24.71 ± 4.81	26.17 ± 6.60	0.49
Duration of illness (years)	8.96 ± 9.72	7.24 ± 6.92	10.00 ± 7.54	0.72
Duration of the prior treatment(years)	8.12 ± 8.83	6.67 ± 7.19	9.34 ± 6.79	0.69
Education (years)	11.91 ± 3.81	12.39 ± 2.46	11.03 ± 3.06	0.14
Alcohol history, N(%)	9/44 (17.0)	4/24 (14.3)	5/25 (16.7)	0.95
Smoking, N(%)	23/30 (43.4)	16/12 (57.1)	15/15 (50.0)	0.39
FGA before screening (FGA/SGA), N(%)	10/43 (18.9)	6/22 (21.4)	7/23 (23.3)	0.79
Family history of schizophrenia, N(%)	17/36 (32.1)	7/21 (25.0)	9/21 (30.0)	0.70

*Abbreviation*: *FGA* First-generation antipsychotics, *SGA* Second-generation antipsychotics

Chi-square test was performed. Unpaired t-test was performed. ^*^p-value< 0.05 statistically significant

**Fig. 1:**
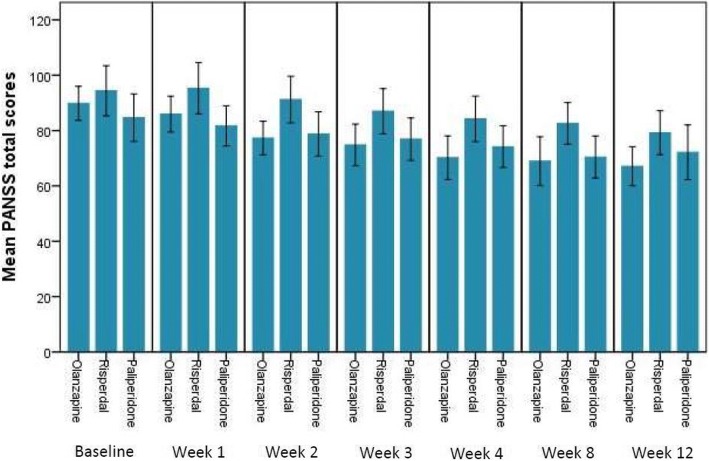
Mean PANSS total scores change over time (PANSS: Positive and Negative Syndrome Scale)

The results showed the mean PANSS total scores changed over time as shown in Table 2 and Fig. 1. Overall, the participants who received either olanzapine, risperidone or paliperidone showed improvement in PANSS rating total scores in each time point. The average PANSS total scores at baseline was 89.59 (SD 22.83), and the clinical response as measured by PANSS total scores was compared among these three treatment groups at all available visits using ANOVA and post-hoc analysis. We found that the comparison of the mean PANSS total scores among these three treatment groups at baseline was not significant (*P* = 0.27) and the decrease in PANSS total scores from baseline was significantly in Olanzapine-treatment group compared to Risperidone-treatment group at Week 2, 4 and 8.

**Table 2:** Mean PANSS total scores change over time (PANSS: Positive and Negative Syndrome Scale) and comparative analysis among group

Mean PANSS total scores	Group variable	N	Mean (SD)	Std. Error	95% Confidence Interval for Mean Lower bound Upper bound		F	*P*
Mean PANSS total scores at baseline (day 0)	Olanzapine	53	89.85 (22.29)	3.06	83.70	95.99		
	Risperidone	28	94.39 (23.40)	4.42	85.32	103.46		
	Paliperidone	30	84.67 (22.99)	4.20	76.08	93.25		
	Total	111	89.59 (22.83)	2.17	85.30	93.89	1.33	0.27
Mean PANSS total scores at Week 1	Olanzapine	45	85.95 (20.88)	3.22	79.45	92.46		
	Risperidone	26	95.31 (22.96)	4.50	86.03	104.58		
	Paliperidone	29	81.69 (19.09)	3.54	74.43	88.95		
	Total	100	87.19 (21.39)	2.17	82.88	91.50	3.03	0.053
Mean PANSS total scores at Week 2	Olanzapine	45	77.29 (20.25)	3.02	71.21	83.37		
	Risperidone	26	91.23 (20.88)	4.09	82.80	99.66		
	Paliperidone	29	78.79 (21.01)	3.90	70.80	86.79		
	Total	100	81.35 (21.27)	2.13	77.13	85.57	4.08	0.02
Mean PANSS total scores at Week 3	Olanzapine	39	74.82 (21.27)	3.72	67.29	82.35		
	Risperidone	26	87.00 (20.19)	3.956	78.85	95.15		
	Paliperidone	29	76.90 (20.21)	3.753	69.21	84.58		
	Total	94	78.83 (21.90)	2.26	74.34	83.32	2.67	0.08
Mean PANSS total scores at Week 4	Olanzapine	37	70.22 (23.62)	3.88	62.34	78.09		
	Risperidone	25	84.24 (19.87)	3.98	76.04	92.44		
	Paliperidone	27	74.19 (19.01)	3.66	66.66	81.71		
	Total	89	75.36 (21.83)	2.31	70.76	79.96	3.30	0.04
Mean PANSS total scores at Week 8	Olanzapine	32	68.94 (24.43)	4.32	60.13	77.74		
	Risperidone	25	82.60 (18.20)	3.64	75.09	90.11		
	Paliperidone	24	70.42 (18.04)	3.68	62.80	78.03		
	Total	81	73.59 (21.47)	2.39	68.84	78.34	3.41	0.04
Mean PANSS total scores at Week 12	Olanzapine	28	67.11 (18.22)	3.44	60.04	74.17		
	Risperidone	25	79.20 (19.26)	3.85	71.25	87.15		
	Paliperidone	18	72.17 (19.90)	4.69	62.27	82.06		
	Total	71	72.65 (19.47)	2.31	68.04	77.26	2.68	0.08

*SD* Standard deviation;^*^p-value< 0.05 statistically significant

**Fig. 2:**
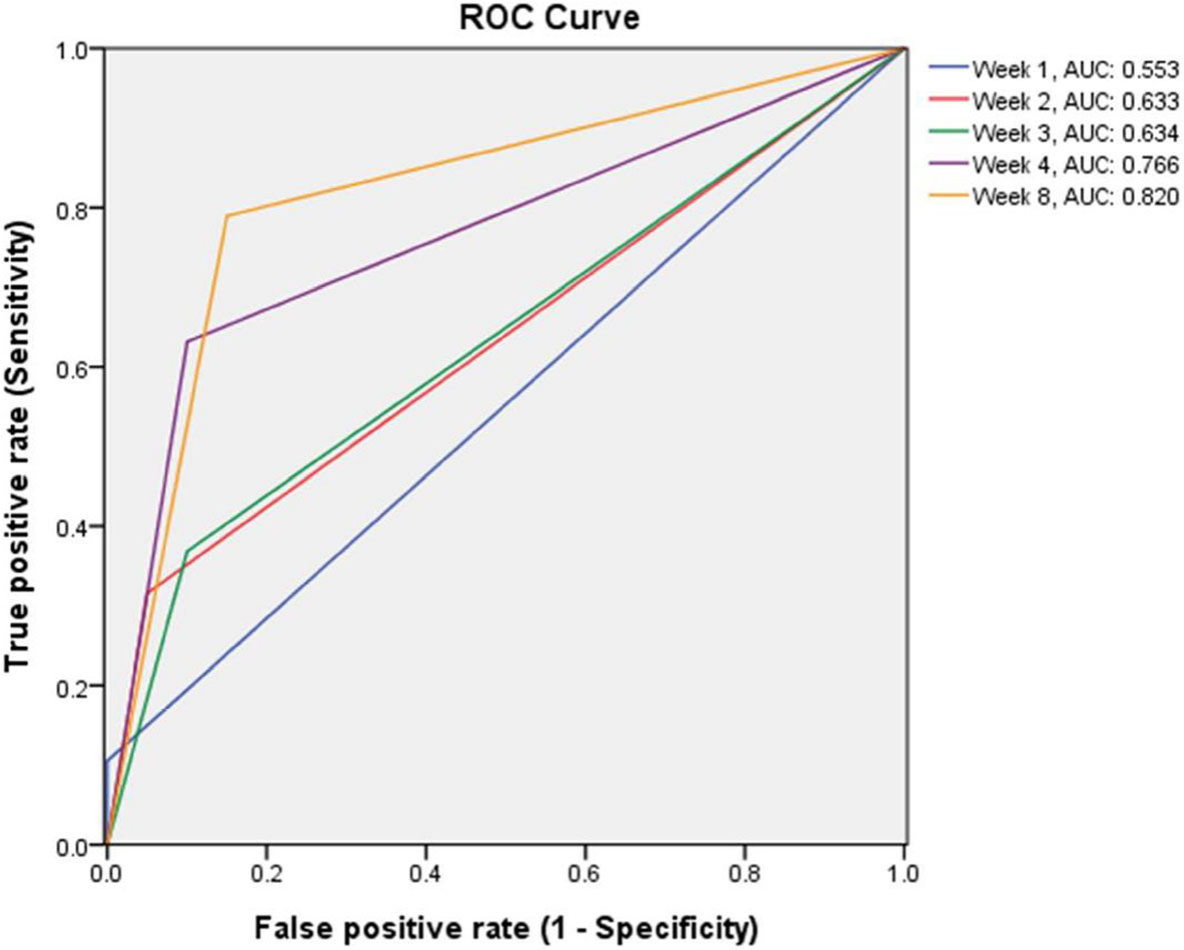
ROC curves of the treatment response at Week 1, Week 2, Week 3, Week 4, and Week 8 for predicting the treatment response at Week 12, when defined as treatment response ⩾25% reduction of the PANSS total score from baseline. AUC: area under the curve; PANSS: Positive and Negative Syndrome Scale; ROC: receiver operating characteristic

Further, we anaylyzed participants with PANSS total score≧25% reduction at five separate study point (Week 1, 2, 3, 4, 8) and used it to identify individuals that are responders or non-responders to the medication therapy. Discrimination of responders from poor responders by weeks are presented in Table 3. By Week 1, 5 of 100 patients (5%) were early-responder; by Week 2, 18 of 100 patients (18%) were early-responder; by Week 3, 21 of 94 patients (22.3%) were responder; by Week 4, 28 of 89 patients (31.5%) were responder and by Week 8, 24 of 81 patients (29.6%) were responder. The sensitivity, specificity, positive predictive value (PPV) and negative predictive value (NPV) were calculated for each response at Week 1, 2, 3, 4 and 8, in terms of predicting thetreatment response at Week 12. We found significant relationships between the early treatment response and ultimate therapeutic outcome since Week 2. At Week 2, a reduction in the PANSS total score of 25% was the best predictor, with a sensitivity of 0.667 and a specificity of 0.721. The PPV was 0.261, the NPV was 0.936 and the accuracy was 0.714. Based on the findings of high NPV (0.936) at Week 2, there was a high probability that continued treatment would not yield further improvement. Thus, the result supports that we may stop the medication for non-responders and consider switching to another antipsychotic drug at Week 2. However, for the predication of ultimate responders, it would be better to predict it until Week 4 (PPV = 0.65) considering relatively lower PPV for Week 1, 2 and 3. The area under the ROC curve (AUC) (Fig. 2) were 0.6 to 0.8, indicating moderate accuracy.

**Table 3:** Conditional probabilities and predictive value of early treatment response at Week 1, Week 2, Week 3, Week 4 and Week 8 predicting ultimate treatment response at Week 12

Predictor Variable ^a^	Early Responder, n (%)	Odds Ratio	*P*	Sensitivity (%)^b^	Specificity (%)^c^	PPV(%)^d^	NPV(%)^e^	Accuracy
Week 1	5/100 (5.0%)	1.21	0.83	9.1%	69.7%	9.1%	69.7%	54.5%
Week 2	18/100 (18.0%)	5.18	0.03	66.7%	72.1%	26.1%	93.6%	71.4%
Week 3	21/94 (22.3%)	5.73	0.01	66.7%	74.1%	34.8%	91.5%	71.4%
Week 4	28/89 (31.5%)	18.11	0.02	76.5%	88.0%	65.0%	90.7%	83.9%
Week 8	24/81 (29.6%)	19.36	0.008	73.9%	87.2%	73.9%	87.2%	82.9%

*PPV* Positive predictive value, *NPV* Negative predictive value

^*^P-value< 0.05 statistically significant

Predictor Variable: ≧25% reduction in PANSS total scores

a Sensitivity x: correct identification of responders, e.g. if 100 patients responded

at endpoint, x (of these 100) patients were correctly identified earlier

b Specificity x: correct identification of non-responders, e.g. if 100 patients failed to respond at endpoint, x (of these 100) patients were correctly identified earlier

c PPV x: proportion of early responders who were responders at endpoint, e.g. if 100 patients were early responders, x (of 100) responded at endpoint

d NPV x: proportion of early non-responders who were non-responders at endpoint, e.g. if 100 patients were early non-responders, x (of 100) failed to respond at endpoint

Potential predictors for ultimate outcome were taken into consideration s in the GEE model. At the end, types of antipsychotic medication, treatment dosage, gender, age, age at onset, duration of mental illness, duration of the prior treatment, educational level, history of alcohol use and smoking, antipsychotics used before screening and family history of schizophrenia were included in the final GEE model. Howevere, there were no statistically significant observed for any predictors (data not shown).

## Discussion

The main finding of this study is that in patients with schizophrenia treated by olanzapine, risperidone or paliperidone the early changes of PANSS total score at Week 2 could predict the ultimate response at Week 12. The early treatment non-response at Week 2 had a greater NPV (93.6%) than did the non-response at Week 1 (NPV, 69.7%), Week 3 (NPV, 91.5%), Week 4 (90.7%) and Week 8 (NPV, 87.2%). In addition, there was no any other potential predictor, including types of antipsychotics and treatment dosage, associated with ultimate response in our study.

These findings demonstrate that in schizophrenic patients treated by olanzapine, risperidone and paliperidone, early non-responders have a very high chance, more than 90%, to become non-responders. Previously, studies on second-generation antipsychotics also found that early improvement of psychotic symptom at Week 2 could predict subsequent treatment responses [1, 2, 5, 7, 9, 11, 16, 18–21]. It has been suggested that a lack of response to antipsychotic agents in the early course can serve as a marker for response defects in patients undergoing treatment [18]. Hence, clinical evaluation of patients during the initial 2-week period might aid the detection of non-responders [18], which is compatible with our finding.

In line with several previous studies on the same type of atypical antipsychotics, our study has similar finding to establish prediction strategy. Stentebjerg-Olesen et al. [21] suggested that schizophrenia treated by olanzapine, early response at Week 2 and 3 could predict ultimate response at Week 6. However, their participants were adolescent with schizophrenia. It may be difficult to generalize to other population. Likewise, previous studies for schizophrenia identified that early psychotic symptom reduction at Week 2 is a suitable predictor for future responses at Week 6 or 12 under the treatment of papliperidone or risperidone [1, 11]. Futhermore, numerous studies have examined the association of NPV values and non-responder in treatment response of schizophrenia and suggested NPV value was greater than PPV [8, 18, 22–24]. These studies also pointed out that for early non-responders of schizophrenic patients, whose NPV values were between 75~ 85%, will most likely continue to be non-responders.

Furthermore, patients with schizophrenia are supposed to exhibit reduced psychotic symptoms after an adequate antipsychotic treatment for 3 to 6 weeks [25]. In our study, the PPV is more acceptable until Week 4 (PPV = 0.65). Based on the evidences above, we recommended that schizophrenic patients treated with olanzapine, risperidone, and paliperidone may consider to switch to another antipsychotic medication if they are non-responders at Week 2, and for those who are responders at Week 2 they should be further observed to see if they are still responders at Week 4 and to decide whether they should keep current antipsychotic or not. According to the study of Leucht et al. [16], the Brief Psychiatric Rating Scale (BPRS) and the PANSS are the most reliable tools for defining the response of a patient to therapy. The reduction of 20 to 50% in the rating scores of the BPRS or PANSS has been considered as treatment response in a number of studies [5, 8, 10, 11]. Our finding is very similar with the study of Yeh et al. [11], who used reduction of 27% on total PANSS score from baseline at Week 2 as a predictor for treatment outcomes at Week 6 with a high statistical power. As previously mentioned, we applied the multiple logistic regression by the GEE analysis to establish a prediction model for the treatment response, defined as a reduction of ≧25% in PANSS total score. However, we did not find any significant associations between predicting variables with treatment outcome of schizophrenia. Prior research has shown that older-onset, shorter duration of illness, and higher education level may play as a predictor in determining good response to conventional antipsychotics and clozapine [26–32]. In risperidone study, patients with a lower educational level has been shown to predict higher symptomology [33]. Considering the inconsistent results, future study with longer observation and larger samples will be a great benefit to explore the possible predictors for the responders in patients with schizophrenis under antipsychotic treatment.

The advantage of our study is that we analysed pooled data of three atypical antipsychotic agents (olanzapine, risperidone and paliperidone) for patients with schizophrenia on the treatment prediction model. We also conducted more frequent assessment time points (Time 0–7), which make us identify the optimal time to decide more accurate treatment strategies (i.e. continuing or switching the medication). Some limitations have to be taken into account before making a conclusion. Firstly, a relatively small sample size and high dropout rate of participants across three treatment groups. Secondly, 7 days’ washout period is not enough and could bias the subsequent treatment. However, it would not be allowed longer-time washout period practically. Thirdly, we should consider to detect plasma level of prescribed drug to determine drug adherence if the participants were treated in outpatient settings. Fourth, the symptomatic improvement is only a part of successful treatment and relevance of other domains of the illness should be considered in defining treatment response in schizophrenia.

## Conclusions

In conclusion, the findings of this study suggest that the revision of treatment strategy should be considered if patients with schizophrenia were not responsive after 2 weeks’ treatment, and for those who are responders at Week 2, another two weeks are needed for further evaluation whether they will be continuously responsive or not. More studies to investigate the other possible predictors are indicated considering the pharmacoeconomics and the sufferings of patients and families.

## Abbreviations

DSM-IV: Diagnostic and Statistical Manual of Mental Disorders, Fourth Edition
GEE: Generalized estimating equations
NPV: Negative predict value
PANSS: The positive and negative syndrome scale
PPV: Positive predict value
